# Lithium Use in a Patient With Bipolar Disorder and End-Stage Kidney Disease on Hemodialysis: A Case Report

**DOI:** 10.3389/fpsyt.2020.00006

**Published:** 2020-02-03

**Authors:** Cheryl W. L. Chang, Cyrus S. H. Ho

**Affiliations:** ^1^ Department of Psychological Medicine, National University Hospital, Singapore, Singapore; ^2^ National Psychiatry Residency Programme, National Healthcare Group, Singapore, Singapore

**Keywords:** lithium, bipolar disorder, chronic kidney disease, end-stage kidney disease, hemodialysis

## Abstract

Lithium has been implicated in causing chronic tubulointerstitial nephritis and decline in renal function. However, lithium still plays a role in stabilizing patients with bipolar disorder. We illustrated a case of a bipolar I disorder patient approaching end-stage kidney disease (ESKD) with other medical comorbidities. As her mental state was affecting her compliance with medical treatment, she was mentally and medically unwell. Our patient was hospitalized on two separate occasions, for 5 and 4 months respectively, and failed adequate trials of different psychotropics due to inefficacy or intolerable side effects. A decision was made between the psychiatrist, nephrologist, and cardiologist to use lithium with hemodialysis support, with good treatment response and improved mental state. This case has shown that lithium carbonate can still be prescribed in ESKD patients on hemodialysis. Daily monitoring of lithium levels in the initial phase of lithium and hemodialysis commencement for at least 2 weeks is imperative, reducing to three times per week pre-hemodialysis towards the end of discharge after three consecutive daily serum lithium levels have stabilized. Clinicians can also consider a target serum level of less than 0.6 mEq/L in maintenance treatment for bipolar disorder in patients on hemodialysis.

## Background

Lithium is one of the first-line treatments for the manic and depressive phases of bipolar disorder (1, 2). It protects against both depression and mania as well as being the only therapy known to reduce suicide risk in this patient population (3–5), with a composite measure of suicide plus deliberate self-harm lowered in patients receiving lithium (5). Even with the availability of newer treatments, lithium is still considered the first-line treatment when prescribing a relapse-prevention drug in bipolar disorder. It has efficacy in the prevention of both manic and depressive episode relapse or recurrence (6), having the most robust evidence for long-term relapse prevention (7).

The exact mechanism of action is unknown, but lithium suppresses inositol signaling through depletion of intracellular inositol and inhibits glycogen synthase kinase-3 (8). Lithium has also been shown to decrease the release of norepinephrine and dopamine from nerve terminals and may transiently increase the release of serotonin (8). In vivo, lithium is distributed in the total body water with minimal protein binding, and the initial distribution is in extracellular fluids, with equilibration into intracellular compartments in 5–10 days (9). Excretion is primarily through the kidneys and urine, with approximately 80% of the lithium that is filtered by the glomerulus being reabsorbed (60% by the proximal tubule and 20% by the thick ascending limb of the loop of Henle and collecting duct) (2, 8). Principal cells of the collecting duct are the primary target for the cytotoxic effects of lithium, which are thought to involve inhibition of signaling pathways that involve glycogen synthase kinase type 3β (10). As a low-molecular-mass monovalent cation that is not metabolized and not protein-bound (9), lithium is easily dialyzable due to its low molecular weight, low protein binding, relatively low volume of distribution, and low endogenous clearance (8).

Lithium has been implicated in causing chronic tubulointerstitial nephritis and about a 30% decline in renal functioning, as measured by the estimated glomerular filtration (eGFR) (11). The duration of lithium prescription and episodes of elevated lithium levels are risk factors predicting the development of lithium-induced nephropathy (1). The absolute risk of renal failure associated with lithium use in those over the age of 50 is small, with a retrospective cohort study showing a 2.3% estimated increase in the risk of renal failure, with a number needed to harm of 44 (12). A review of literature on the use of lithium in older patients showed that older lithium users commonly have chronic kidney disease (CKD), although rates are not different compared to community-dwelling non-lithium older patients (13). There are also concerns that lithium nephropathy may continue to progress despite the cessation of lithium (1).

However, a Denmark cohort study of the lithium continuation versus other anticonvulsants in patients with bipolar disorder after diagnosis of CKD showed that continuing lithium was associated with decreased end-stage kidney disease (ESKD), as compared to other anticonvulsants (14). Switching to another anticonvulsant did not yield any advantage (14). The data on the development of ESKD are diverse, ranging from 0.53% in earlier studies to no difference in prevalence in recent studies (15).

Hence, lithium still plays a role in stabilizing bipolar disorder if other trials of psychotropics fail. Reports have proposed the appropriate usage of lithium in older patients with CKD, which requires judicial administration and monitoring. It is recommended that renal function and serum lithium (keep level <0.8 mmol/L) monitoring should be done every 3 to 6 months (13). Nevertheless, there are no specific guidelines on dosing and monitoring of lithium in bipolar disorder patients with ESKD on hemodialysis.

## Case Presentation

A 56-year-old Chinese woman diagnosed with bipolar I disorder for the past 28 years was transferred from a tertiary psychiatric hospital in 2017 to our medical hospital for bacterial endophthalmitis. She was also experiencing a manic relapse concurrently, presenting with elated and labile mood, irritability, flight of ideas, disinhibition, and grandiose delusions of being a beauty pageant contestant. Her mental state affected her insight into and compliance with all medical treatment, and as such, she was mentally and medically unwell.

Her bipolar disorder was previously stabilized on dual mood stabilizers (lithium carbonate at 400 mg per day, and sodium valproate at 1,000 mg per day). Since the diagnosis of her medical comorbidities in 2010, including CKD approaching ESKD secondary to her poorly controlled diabetes mellitus and hypertension, hyperlipidemia, and obstructive sleep apnea, her lithium was ceased due to CKD, switching to antipsychotics (including sulpiride at 400 mg per day, and risperidone at 4 mg per day) and sodium valproate at 1,200 mg per day. However, she continued to have multiple relapses, necessitating three admissions between the years of 2011–2014 for mania. Despite inpatient treatments, her condition was not stabilized, and she was trialed on intramuscular antipsychotics (zuclopenthixol at 400 mg every 4 weeks) due to poor compliance with oral medications.

Our patient was first hospitalized in our medical hospital for 5 months, complicated by five intensive care unit admissions due to decompensated type 2 respiratory failure. Although there were initial improvements in the mental state with sodium valproate and risperidone, she developed bicytopenia on sodium valproate, and had repeated episodes of drowsiness, prolonged corrected QT interval (QTc), and bradycardia likely secondary to antipsychotics. Electroconvulsive therapy (ECT) was considered, but the patient was deemed to be of high general anesthetic risk. She also had a permanent pacemaker implanted after a diagnosis of sick sinus syndrome by the cardiologist. She was then discharged to the rehabilitation hospital with aripiprazole at 10 mg per day but continued to display residual manic symptoms, including irritability and distractibility. She was admitted again 2 months after her discharge from the rehabilitation hospital for a relapse of manic symptoms, presenting with irritability, elated and labile mood, poor sleep, flight of ideas, and pressured speech. She also had persistent diarrhea and poor management of her fluid overload.

## Description of Laboratory Investigations and Diagnostic Tests

During the second admission, she was trialed on haloperidol with minimal efficacy and problematic side effects (prolonged QTc), and sodium valproate (once again complicated by thrombocytopenia and refusal of blood monitoring). She was admitted to the intensive care unit again due to ventricular fibrillation secondary to prolonged QTc, progressive CKD with eGFR <10, and recurrent hypoxic events.

In view of her thrombocytopenia and ateriovenous fistula (initially electively created in view of progressive CKD) in her left upper limb, intramuscular rapid tranquilization and physical restraint became challenging. ECT was still considered a procedure with a high mortality risk due to her medical comorbidities. Considering her worsening anasarca, anuria, and poor compliance with both medical and psychiatric treatment, her cardiologist, nephrologist, and psychiatrist were in agreement in commencing lithium carbonate controlled-release (CR), with hemodialysis initiation and olanzapine at 10 mg per day.

**Figure 1:**
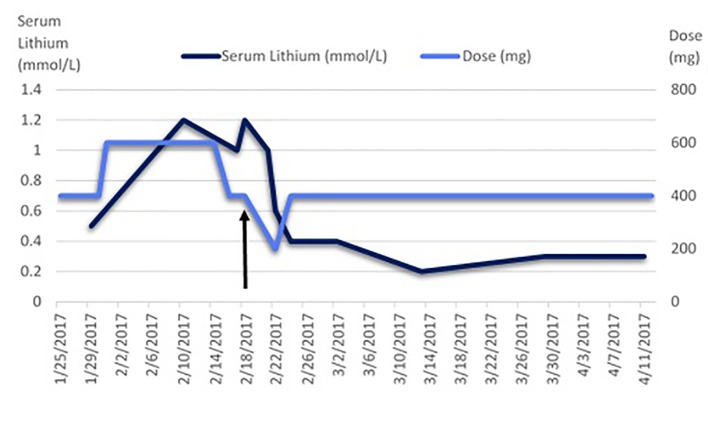
Serum lithium levels measured while on lithium after the commencement of hemodialysis. The arrow indicates the transition of the hemodialysis schedule and lithium dosing from daily to thrice per week. The normal reference range is indicated as 0.6–1.2 mmol/L.

During the initiation of hemodialysis, our patient's fluid status was not stabilized, and she was not compliant to dry weight checks after dialysis, making a clinical assessment of her fluid status challenging. Her lithium levels were monitored closely with pre-dialysis blood sampling before each hemodialysis session. As seen in **Figure 1**, her lithium levels were fluctuating from 0.5 to 1.2 mmol/L when she was prescribed with 400–600 mg per day of lithium carbonate CR while on erratic hemodialysis sessions, although symptoms of lithium toxicity were not clinically present. With close liaison with the nephrologist, the psychiatric team decided to reduce the lithium dosing to post-dialysis doses of 400 mg thrice per week, complementing her hemodialysis scheduling. With the stabilization of her dry weight over the weeks, it was observed that her lithium levels stabilized at 0.3 to 0.4 mmol/L, with good symptoms resolution. She was discharged after a 4-month extended stay, with her manic symptoms remitting 6 weeks after initiation of lithium. She continued to maintain a stable mental state while on lithium 400 mg three times per week 2 years after her discharge.

## Discussion

In the case discussed in this paper, the patient presented with a clear need to reconsider lithium in treating her bipolar disorder. Our medical hospital provided collaborative care for this complicated case and proved that lithium can still be considered for ESKD patients (2, 16, 17). There were no apparent medical or psychiatric benefits in stopping lithium treatment; rather, this aggravated her psychiatric condition. The extended-release formulation of lithium was used in this patient, and this is recommended, as it potentially minimizes the risk of side effects and toxicity. Although no guidelines were found to guide our management (2), there have been a few cases reported with the successful usage of lithium in ESKD patients dosed immediately post-dialysis (17, 18). Subsequently, we dosed lithium in the evenings after her scheduled hemodialysis, effectively dosing her thrice per week, with serum lithium levels stabilizing at 0.3 mmol/L.

We initially monitored her serum lithium levels every 3 days and saw that levels changed drastically from 0.5 to 1.2 mmol/L in 12 days, with fluctuations between 1.0 and 1.2 mmol/L when lithium was kept constant at 600 mg daily, together with daily hemodialysis. However, we kept in mind that her hemodialysis schedule was not stabilized as she was receiving ad-hoc hemodialysis due to her anasarca. Therefore, we recognized that the lithium serum levels would not be useful to titrate her lithium dosing regime, although they could be used to monitor for toxicity, as CKD patients are particularly prone to lithium toxicity (18–20), with a propensity of lithium to accumulate (21). Moreover, it is believed that the fluctuation of serum lithium levels is the result of re-equilibration from the intracellular space following clearance of lithium from the extracellular space during dialysis (9, 22). It would be worthwhile to obtain residual trough serum lithium levels right before hemodialysis in order to establish the fluctuation of serum levels, which was not well established during hemodialysis. However, we were unable to obtain this information. We suggest that daily monitoring of lithium levels in the initial phase of lithium and hemodialysis commencement for at least 2 weeks is imperative, reducing to three times per week pre-hemodialysis towards the end of discharge after three consecutive daily serum lithium levels have stabilized. Subsequently, 3-monthly to yearly monitoring of serum lithium levels may be considered (2).

We also suggest that clinicians consider a lower target for serum lithium levels in maintenance treatment for bipolar disorder in ESKD patients on hemodialysis. Although there are recommendations to suggest 0.6 to 0.8 mEq/L as target levels (18, 20), our case illustrated that the initial serum lithium levels above 0.8 mmol/L helped treat her manic symptoms acutely. Subsequently, with lower serum lithium levels of 0.3 mmol/L, she continued to demonstrate remittance of her manic symptoms. Hence, this may suggest that such patients who respond well to lithium need not have high serum lithium levels during maintenance treatment, especially with ESKD and the higher risks of lithium toxicity.

## Concluding Remarks

This case report has shown that lithium carbonate CR can still be prescribed for patients with ESKD, with close monitoring to determine safety, dosage, and monitoring of lithium. The benefits of lithium treatment in her case outweighed the risks involved. Proposed guidelines to monitor lithium use in chronic renal disease patients should also be developed, as we believe more of such cases will appear with our ageing population. The unique inpatient facility in our hospital was also imperative in providing optimal collaborative care with psychiatrists, nephrologists, and cardiologists for this particular group of patients. Analyzing the serum lithium levels before hemodialysis may be helpful in the future to further our understanding of the fluctuations seen in such patients.

## Data Availability Statement

All datasets generated for this study are included in the article/supplementary material.

## Ethics Statement

Written informed consent was obtained from the individual(s) for the publication of any potentially identifiable images or data included in this article.

## Author Contributions

CC and CH jointly wrote the original draft and amended the manuscript.

## Conflict of Interest

The authors declare that the research was conducted in the absence of any commercial or financial relationships that could be construed as a potential conflict of interest.
